# Are Councils on Chiropractic Education expectations of chiropractic graduates changing for the better: a comparison of similarities and differences of the graduate competencies of the Chiropractic Council on Education-Australasia from 2009 to 2017

**DOI:** 10.1186/s12998-020-00315-8

**Published:** 2020-05-24

**Authors:** Stanley I. Innes, Amanda Kimpton

**Affiliations:** 1 College of Science, Health, Engineering and Education, Murdoch University, Murdoch, Australia; 2 School of Health and Biomedical Sciences, RMIT University, Melbourne, Australia

**Keywords:** Accreditation, Chiropractic, Education, Standards

## Abstract

**Background:**

The Council on Chiropractic Education Australasia (CCE-A) is tasked with assessment and accreditation of chiropractic programs (CPs) in the Australasian community. To achieve this process the CCE-A has developed educational standards and graduate competencies which include minimum expectations of graduates prior to entry into the workforce. We sought to explore if these are changing overtime, and if so are these changes for the better.

**Method:**

The CCE-A 2009 and 2017 Competency Standards were located and downloaded. The competencies were placed into tables for a comparative analyses in a systematic manner to enable the identification of similarities and differences. In addition, word counts were conducted for the most commonly occurring words and this took place in December 2019.

**Results:**

The 2017 competency standards were over three times smaller than the previous standards 2009 standards. More similarities than differences between the old and the new standards were found. There were 18 additions to the 2017 graduate competencies with many that were in unison with contemporary aspects of healthcare such as patient centred-care, respect for practitioner-patient boundaries and patient sexual orientation, transitioning patients to self-management, and consideration of improving lifestyle options. Some competencies were not bought forward to the new standards and included, among others, students being competent in screening for mental health conditions, an expectation to discuss cost of care, re-evaluating and monitoring patients at each visit, and knowing when to discharge patients. The competencies continued to be silent on known issues within the chiropractic profession of a lack of a definition for chiropractic that would inform scope of practice and the presence of vitalism within CPs.

**Conclusion:**

There have been positive changes which reflect contemporary mainstream health care standards between CCE-A graduate competency revisions. The absence of a clear definition of chiropractic and its attendant scope of practice as well as continued silence on vitalism reflect known issues within the chiropractic profession. Recommendations are made for future accreditation standards to inform the required competencies and aid the integration of chiropractic into the broader health care community.

## Background

The economic reality of the impost that musculoskeletal related pain and disability places on economies [1] has resulted in a calls for changes in management [2]. This problem has grown in magnitude over several decades and it is logical to assume that governments and the health care sector would respond to this concern by making changes overtime. One possible strategy that governments could use where changes overtime may be evident is the accreditation of health training programs [3, 4]. This is thought to be one of many levers to stimulate systems-level improvement by promoting uptake of optimal, evidence-based governance and clinical standards [5].

Accreditation is undertaken for chiropractic programs by the Councils on Chiropractic Education (CCEs). Such CCEs are found in Australia (CCE-A) [6], Canada (CCE-Canada) [7], Europe (European-CCE) [8] and in the United States of America (CCE-USA) [9]. Each of the CCEs have developed a list of the minimum expectations for educational standards and competencies expected of graduates before they enter the workplace. The graduate competencies are a list of descriptive statements to clarify the necessary knowledge, understanding, skills, attitudes, and behaviours students should attain before entering practice [10]. The attainment of the set competencies is intended to ultimately improve the quality of societal levels of health care and patient safety.

### Problem

The literature surrounding the practice of chiropractic indicates the most common clinical encounters are musculoskeletal in nature, especially of the spine [11], and that there is a desire from some for the profession to be viewed by the broader community as experts in conservative spine care [12, 13]. However, there is a growing body of evidence of persistent patterns of undesirable care within chiropractic practice that mainstream allied health care would not accept as best quality, including but not limited to, anti-vaccination beliefs, non-guideline X-ray usage, low levels of interdisciplinary interaction, and excessive non-indicated care [14–16]. These patterns have been attributed to chiropractors who adopt a subluxation or vitalist model [14]. Agencies or regulators with the responsibility to optimise the quality of chiropractic education are expected to revise their standards from time to time. It seems reasonable that CCE documentation relating to accreditation standards and processes would reflect known problems as they seek to produce graduates more able to deal with the working world they are entering. In particular, with the ever-increasing burden of musculoskeletal related pain and disability, graduates equipped with skills to deliver care in a way that is socially responsible and, in the public’s best interests [17]. However, this may not be the case [18–21].

While revisions of the CCE-International accreditation standards for chiropractic programs have been reviewed for changes over time [22] no study has explored expectations for graduating chiropractors. Consequently, we chose to investigate the graduate competencies of one CCE, namely the CCE-A for such changes.

### Objectives

The objectives of this study were (i) to compare the CCE-A 2017 Competency Standards with their previous 2009 Competency Standards, to explore similarities and differences of prescribed recommendations and (ii) to comment on whether these changes are likely to be for the better or the worse (iii) If possible, make recommendations for improvement.

## Methods

We conducted a systematic investigation into the first two objectives. This initially involved a comparison of the content of the CCE-A Graduate Competencies from 2009 and 2017 looking for similarities and differences. As part of the analysis we included 100 words and compared them for increased or decreased frequency of usage as well as words that had been deemed to be important indicators for recognising change in CCE documentation in previous similar research [22].

### Data extraction process and synthesis of results

The CCE-A website was searched for the current Accreditation and Educational Competency Standards and the current Framework for Chiropractic Education and Accreditation was downloaded in June 2019 [6]. The publication date was identified as 2017. A web library [23] was used to search CCE-A website history to find information about the date for the previous standards, which was found to be December 2009. This matched information used in a prior study [24].

The PDF texts of the downloaded 2009 and 2017 CCE-A graduate competencies were converted to Microsoft Word format. The Word documents were compared to the PDF texts to ensure that no errors had occurred. The 2017 competencies were placed into a table according to their published structure of five competencies: Practicing Professionally, Communication Collaboration and Leadership, Clinical Assessment, Planning Care and Implementing Monitoring and Evaluating Care. Each of the competencies is supported by varying un-numbered descriptive statements called “Performance Criteria”. For example, Competency Three in the 2017 graduate competencies is Clinical Assessment and has five subdomains. The first is numbered as “3.1. Obtains and records a history”. This is accompanied by three performance criteria intended to more fully describe or inform the chiropractic program (CP) of the expectations by the CCE-A. We numbered these according to their printed order. Thus, the first was designated “3.1.1 Obtains and records history of patients’ medical, social and health status”. We also numbered the 2009 graduate competencies in this way.

A table was constructed using the CCE-A 2017 graduate competencies. There were five competencies and a total of 22 subdomains. Each author then independently sought to place or match each statement in the 2009 standards with a comparable one in the 2017 standards. This resulted in the construction of a table where a visual inspection could identify similarities and differences. The authors then compared their findings and discussed any differences. A third author was available to resolve any differences that could not be resolved.

In the second phase a comparison of contents was made by counting words in the two documents. The “Appendix” section and the “Accreditation Standards for Chiropractic Programs” of the 2017 standards was not included in the word count as there was no equivalent section in the 2009 standards and it only contained definitions of words and the rationale for their use. Content analysis using word counting is widely used in qualitative research [25–27]. A summative content analysis involves reading the data several times for familiarisation to provide the opportunity to reflect on the overall meaning. The data was then coded and compared, usually for keywords or content and generally tabulated [27]. This process was used to facilitate the subsequent interpretation of the underlying context and has been used in previous research exploring changes in CCE accreditation standards over time [22].

After this process, the lead researcher identified 100 predominately adjectival words, seen in Table 3, considered to reflect the content and intent of the educational standards. These words related to the practice of chiropractic as well as the assessment of chiropractic practice. The lead author (SI) then searched for each word using the ‘Find’ function in Microsoft Word. All occurrences of the word were copied verbatim, including the sentence in which it was found so it could be seen in its context, and listed in a spreadsheet. The final list was reviewed and discussed with the other author (AK).

The second phase of the investigation determined the frequency of the use of each word and whether it was being directed toward the student or was being used as a heading (in larger font or bold indicating a section of information) or if it had another unrelated purpose. The context or intent of the use was then determined by following the categorization of ‘heading’ or ‘text’. For example, the word ‘collaboration’ was searched for in the 2017 standards. It occurred as a heading in the Universal Competency 2 Communication, Collaboration and Leadership and as text as expectation that a graduate will be able to “communicate and collaborates effectively at all times with patients and others”. Uncertainty over the intent of any word was discussed with the second author (AK). Any disagreement between the two authors was resolved by discussion with another researcher experienced in this area.

In the final part of this phase, the extracted spreadsheet was visually examined for an increased, decreased or unchanged frequency of the occurrence of the words when compared across CCE-A competencies standards for 2017 and 2009. Consideration was given to the 2009 standards being 2009 approximately 3 times larger than the 2017 standards.

## Results

There was a high degree of agreement between the two researchers on the classification of the similarities and differences and the context of the prescribed key words. The third researcher was therefore not required to resolve any disagreements.

### General impressions

The 2017 CCE-A Competency Standards when compared to the 2009 Standards are considerably smaller (Table 1). This is evidenced by the 2017 Standards containing approximately 1/3 the number of words than the 2009 standards (not including Foreword/Introduction sections), half the Graduate Competencies / Domains, 1/3 less sub-competencies/sub-domains and almost 4 times less Performance Criteria.

**Table 1:** Comparison of CCEA 2009 and CCEA 2017 graduate competencies structure

	CCEA 2017	CCEA 2009
**Word Count**	1729	4935
**Competencies**	5	11
**Sub-competencies**	22	38
**Performance Criteria**	71	269

The 2017 Standards contain a Glossary of Terms with definitions of 14 words and terms as compared to a smaller list of 10 in the 2009 Standards.

Neither the 2009 or 2017 competencies for graduating chiropractors contained a definition of chiropractic or a chiropractor.

### Method of development of standards

The 2017 Standards were based on the Australian Dental Council research and accreditation standards. In addition, an “environmental scan of the current research, policy and practice in health, education, the profession and regulation in Australia and New Zealand and other international loci” was included. From this a Consultation Paper was prepared that then underwent two rounds of consultation with stakeholders. The Steering Committee membership and qualifications are listed. This information is not provided in the 2009 Standards.

### Graduate competencies

There were many more similarities than differences when comparing the graduate competencies of the CCE-A of 2017 and 2009 and are found in detail (Table 2) and in a summary format (Table 3).

**Table 2:** Comparison of CCEA 2009 and 2017 graduate competencies

CCE-A 2017 competency		Description (Denotes only present in 2017)	CCE-A 2009 Equivalent
1	PRACTICING PROFESSIONALLY	Practises professionally, ethically and legally with safety and efficacy with the application of evidence-based practice as the primary consideration in all aspects of chiropractic practice.	1.1
1.1	Complies with legal and ethical requirements		
1.1.1		Adheres to relevant legislation, common law, codes, standards and other policy regulating chiropractic conduct and practice.	1.1/ 3.4/ 4.1/ 4.2 / 5.1
1.1.2		Applies the ethical principles of autonomy, beneficence, nonmalfeasance and justice.	1.1
1.1.3		Applies principles of *confidentiality* and privacy.	9.2/ 9.3
1.1.4		Establishes and maintains professional relationships and *boundaries.*	3.3/ 9.4
1.2	Applies a patient-centred approach to practice		
1.2.1		Recognises and responds to diversity in the population, including but not limited to gender, age, religion, race, disability, socioeconomic status and *sexual orientation.*	6.1/ 9.3/ 10.1
1.2.2		Recognises and responds to the impact of culture, values, beliefs, education levels and life experiences on health status, health and help-seeking behaviours and maintenance of health.	6.1/ 9.3/ 10.1
1.2.3		Recognises and responds to a patient’s emotional response to their health status.	6.3B
1.3	Applies an evidence-based approach to practice		11.1
1.3.1		Uses an evidence-based approach in planning, delivering and evaluating care.	11.1
1.3.2		Applies critical thinking and problem solving to all aspects of care.	11.1
1.4	Demonstrates professional integrity		3.2
1.4.1		Demonstrates commitment to continuing professional development and lifelong learning.	3.2
1.4.2		Applies research skills to support professional development and lifelong learning.	11.1
1.4.3		Works within the bounds of their professional expertise and competence and *seeks professional support and peer review when necessary.*	3.4/ 6.5/ 8.4
1.4.4		Accepts responsibility and accountability as a professional and member of the chiropractic profession.	3.4
1.4.5		Applies principles of risk management and *quality improvement to practice.*	6.4/ 8.4
1.5	Demonstrates capacity for self-reflection		3.2
1.5.1		Demonstrates skill in self-assessment and critical evaluation of personal knowledge, skills and expertise, including awareness *of personal bias* and beliefs and how these might influence patient care; has appropriate strategies in place to deal with this.	3.2/ 6.5/ 8.4
1.5.2		Demonstrates awareness of factors affecting their health and wellbeing, including fatigue, stress management, infection control and disease prevention, to mitigate health risks of professional practice	
2	COMMUNICATION, COLLABORATION AND LEADERSHIP	Communicates and collaborates effectively at all times with patients and others	6.1/ 6.2/ 6.5/ 6.6/ 6.7/ 7.1/ 7.2/ 9.1/ 9.2/ 9.3/ 9.4/ 9.5
2.1	Communicates effectively with patients and others		9.1/ 9.2/ 9.3/ 9.4
2.1.1		Communicates effectively – verbally, non-verbally and in writing – providing clarity for safe and agreed care and treatment.	6.1/ 6.2/ 9.1/ 9.2
2.1.2		Meets language proficiency requirements established in regulation for the profession.	
2.1.3		Adapts communication style to acknowledge cultural safety, and cultural and linguistic diversity	6.1/ 9.4
2.1.4		Uses information and communications technology effectively to enhance communication	
2.2	Collaborates effectively with patients and others		
2.2.1		Demonstrates rapport, active listening, mutual respect and trust in developing professional relationships with patients and others.	9.4/ 9.5/ 6.1/ 6.2
2.2.2		Expresses professional opinions competently, confidently and respectfully, *avoiding discipline specific language when necessary.*	9.5
2.2.3		Gives *timely,* sensitive and instructive feedback to colleagues in the chiropractic profession and other professions and responds professionally to feedback from these colleagues.	3.3/ 9.4
2.2.4		Demonstrates ability to describe and respect the roles and expertise of other health care professionals.	2.1 / 3.2/ 3.3
2.2.5		Demonstrates ability to learn and work effectively as a member of an inter-professional team or other professional group, including through delegation, supervision, consultation and referrals.	2.1/ 3.3/ 9.4
2.2.6		Recognises potential for disagreement and conflict in relation to care and management, and responds to resolve issues	
2.3	Collaborates effectively with patients and others		
2.3.1		Recognises responsibility to protect and advance the health and wellbeing of individuals, communities and populations.	1.1/ 10.1
2.3.2		Participates in evidence-based health education and risk reduction programs to meet identified needs within the community.	1.2/ 11.1
2.3.3		Integrates prevention, early detection, health maintenance and *chronic condition management,* where relevant, into practice.	10.1
2.3.4		Places the needs and safety of patient*s at the centre of the care process,* demonstrating safety skills including infection control, adverse event reporting and effective co-management and referral.	1.2/ 9.10/ 10.1
2.4	Manages information to meet legal obligations and professional standards		
2.4.1		Creates, maintains and manages accurate and complete records that comply with legal requirements, accepted professional standards and confidentiality	4.1
2.5	Supervises administrative and other staff		
2.5.1		Defines activities that can be delegated to administrative or other staff.	4.2
2.5.2		Explains responsibility for supervising and training administrative or other staff	4.2
3 CLINICAL ASSESSMENT		Understands patients’ health status and related circumstances; critically analyses these and forms a clinical impression.	
3.1	Obtains and records a history		
		Obtains and records history of patients’ medical, social and health status.	6.1
		Evaluates individual patient risk factors.	6.1
		Maintains secure, accurate, consistent, *legible* and contemporaneous records of patient management— *electronic* and/or written.	4.1
3.2	Performs a clinical examination		
3.2.1		Explains need for and process of examination.	6.2
3.2.2		Performs examinations relevant to patients’ presentation.	6.2/ 6.3A,B/ 6.4
3.2.3		Obtains consent and conducts physical examination with appropriate rapport, respect and preservation of modesty.	6.2
3.3	Obtains the results of clinical, laboratory and other diagnostic procedures necessary to inform care		
3.3.1		Identifies existing investigation results and reports.	6.5
3.3.2		Determines clinical, laboratory and other diagnostic procedures relevant to patients’ presentation	6.4 / 6.5
3.3.3		Refers for or conducts imaging where clinically indicated.	6.4
3.3.4		Makes referrals or obtains further information, where indicated.	7.2
3.4	Recognises determinants of health		
3.4.1		Identifies and considers determinants of health, including psychological, biological, social, cultural, environmental, educational, and economic determinants, as well as health-care system factors.	1.2
3.4.2		Demonstrates knowledge of aetiology, pathology, clinical features, natural history and prognosis for common and important presentations.	1.2/7.1
3.4.3		Recognises and responds to public health priorities.	1.2
3.5	Critically analyses information available to generate a clinical impression		7.1
3.5.1		Demonstrates knowledge of diagnostic imaging techniques and procedures, including indications and limitations of available imaging modalities.	6.4
3.5.2		Interprets and integrates results of clinical, laboratory and diagnostic procedures into care planning.	6.2/ 6.6
3.5.3		Forms an understanding of patients’ health status and/or identifies possible diagnoses.	7.1
3.5.4		Identifies *‘red flags’* and manages and/or refers as appropriate.	7.2/ 9.8
4	PLANNING CARE	Works in collaboration with patients, exploring the care options available and developing agreed, evidence-based care and management plans.	7.2
4.1	Identifies possible care and management options		
4.1.1		Integrates knowledge of chiropractic and other health sciences to inform decisions about care and management options.	8.1 / 8.2
4.1.2		Obtains, interprets and applies current evidence and information to inform decisions about care and management options.	
4.1.3		Identifies care and management options likely to be therapeutically effective and safe for patients	8.4/ 9.2
4.1.4		Adapts practice according to varying patient needs across the human lifespan, including need for care and management options to be tailored for patients.	1.2
4.1.5		Considers opportunities to enhance patients’ care and management through the involvement of other health professionals.	7.2/ 9.4/ 9.6
4.2	Discusses care and management options		
4.2.1		Explains and discusses the outcomes and implications of the clinical assessment with the patients.	6.5 / 9.1
4.2.2		Discusses purpose, nature, benefits, risks and expected outcomes of care and management with patients and others.	9.1/ 9.4/ 9.6
4.2.3		Discusses and seeks agreement with patients and others on patients’ goals and priorities.	9.3
4.2.4		Describes areas of practice of other health professions and explains interprofessional approaches to patients and others.	9.4
4.3	Formulates a care and management plan		
4.3.1		Formulates care plan in *collaboration* with patients, recognising personal and professional limitation	3.4/ 8.4/ 9.6
4.3.2		Reaches agreement on patient-centred, evidence-based care plan, including chiropractic care, co-management or referral.	
4.3.3		Establishes plans for review of care and management.	8.3/ 9.10
5	IMPLEMENTING, MONITORING AND EVALUATING CARE	Coordinates the safe and effective implementation, monitoring and evaluation of patients’ care and management plans.	
5.1	Obtains and records patient-informed consent regarding care		4.1
5.1.1		Applies relevant legal requirements, professional standards & codes to obtain & record patients’ consents.	3.3
5.2	Implements interventions safely and effectively		
5.2.1		Performs safe and effective adjustive, manipulative, manual and other procedures.	9.6/ 9.8
5.2.2		Provides information and advice to patients for health promotion, *self-management and lifestyle options for better health.*	1.2/ 10.1
5.2.3		Adapts interventions accounting for factors such as age, condition, health status, response to care and patients’ preferences.	1.2/ 9.8
5.3	Monitors & evaluates progress of care and health outcomes		
5.3.1		Recognises possible complications/adverse events arising from patients’ management and has appropriate procedures in place in order to be able to effectively manage these including referral for emergency care when appropriate.	7.2/ 9.7
5.3.2		Monitors patients’ progress towards achieving planned health outcomes *using valid and reliable measures where available.*	7.2/ 9.10
5.3.3		Monitors management and care for adverse events and changes in patients’ lives that may affect care.	7.2/ 9.7/ 9.10
5.3.4		Considers alternative options when indicated.	9.9/ 9.10
5.4	Adapts plans based on monitoring and evaluation		
5.4.1		Collaborates with patients and other health professionals, where indicated, to address issues arising from monitoring and evaluation.	9.10

**Table 3:** A summary of criteria found in the CCE-A 2017 graduate competencies but not in 2009, criteria not carried forward, and those omitted from both

**Criteria present in CCE-A 2017 and not found in 2009 competency standards for graduating chiropractors**	
• Applies principles of *confidentiality* and privacy.	
• Establishes and maintains professional relationships and *boundaries.*	
• Recognises and responds to diversity in the population, including but not limited to gender, age, religion, race, disability, socioeconomic status and *sexual orientation.*	
• Works within the bounds of their professional expertise and competence and *seeks professional support and peer review when necessary.*	
• Applies principles of risk management and *quality improvement to practice.*	
• Demonstrates skill in self-assessment and critical evaluation of personal knowledge, skills and expertise, including awareness of *personal bias* and beliefs and how these might influence patient care; has appropriate strategies in place to deal with this.	
• Demonstrates awareness of factors affecting their health and wellbeing, including fatigue, stress management, infection control and disease prevention, to mitigate health risks of professional practice	
• Meets language proficiency requirements established in regulation for the profession.	
• Added use of information and communications technology effectively to enhance communications	
• Expresses professional opinions competently, confidently and respectfully, *avoiding discipline specific language when necessary.*	
• Gives *timely,* sensitive and instructive feedback to colleagues	
• Recognises potential for disagreement and conflict in relation to care and management, and responds to resolve issues	
• Integrates prevention, early detection, health maintenance and *chronic condition management,* where relevant, into practice.	
• Places the needs and safety of patients at the centre of the care process, demonstrating safety skills including infection control, adverse event reporting and effective co-management and referral.	
• Reaches agreement on patient-centred, evidence-based care plan, including chiropractic care, co-management or referral.	
• Provides information and advice to patients for health promotion, *self-management and lifestyle options for better health.*	
• Monitors patients’ progress towards achieving planned health outcomes using valid and reliable measures where available (previously used pre-determined decision points to re-evaluate).	
• Identifies *‘red flags’* and manages and/or refers as appropriate.	
**Criteria not carried forward to CCE-A 2017 that are present in 2009 competency standards for graduating chiropractors.**	
• Awareness of professional special characteristics, aspirations and strengths (ethos), aware of local to international organisations and major historical mileposts (3.1).	
• Discuss with patient (9.1)	
◦ cost of care	
◦ appropriate patient discharge,	
• Understands relevant health care economies (2.2)	
• Patient re-evaluation and monitoring time frame removed at each visit (9.10)	
• Change in language from “differential diagnosis” to “clinical impression”	
• Removal of chiropractic techniques and replaced with “adjustive, manipulative, manual and other therapies”	
• Removal of must have a “rational for treatment”	
• Removal of must know “contra / non / indications” for care. Replaced with “therapeutically effective”	
• Competent in business, staff and financial management (4.1, 4.2)	
• Requirement to adhere to major national professional organisation (ACA / CA?)	
• An interim management plan is required (8.2)	
• Managing the physical and psychological practice environment (5).	
• Identifies & uses screening instruments for the most common mental health &/or psychological disorders (6.1)	
• Discussion of radiographic technology (6.4)	
• Abnormal physical findings are pursued & investigated in a deliberate, logical & appropriate manner (6.2)	
• The reliability of the data obtained is assessed & appropriate correlation with that patient’s complaints is established where possible (6.2)	
• Patients are appropriately referred to mental health professionals (7.2)	
• Reference to practice furniture, colour coding, music, temperature training of staff “to maintain an environment of unconditional positive regard” (5.1)	
• Financial management of practice (4.1)	
• Ensures adequate, ongoing care for patients during times of absence (3.4)	
• Demonstrates willingness & capacity for writing third party & medicolegal reports certificates & correspondence (3.4)	
• Demonstrates the ability to measure impairment, disability & handicap (3.4)	
**Missing from both the CCE-A 2009 and 2017 graduate competency standards**	
Vitalism / subluxation discussion	
A definition of chiropractic or chiropractor	

#### Similarities

Almost all domains and subdomains in the 2009 graduate competencies could be matched to the 2017 standards of the CCE-A. Both standards included the “Professional domain” and “Practitioner-patient interface domain”. The later encompasses the chronological order of a patient interaction with a chiropractor for a competent clinical assessment, followed by planning and implementing care.

The “professional domain” related to practitioner performance expectations for their fiduciary duties of professionalism, integrity, ethical practice, beneficence, and non-malfeasance. Further there is the expectation of being a life-long learner working collaboratively within the health care system promoting public health.

Some of these standards were not at the same level. For example, the 2017 standards placed the competency of communication at a domain level while it was placed at the sub-domain of “performance indicators” level within the 2009 standards.

#### Differences

The 2009 standards number of domains / competencies have been increased from four to five. The 2009 domains of “The community” and “Professional management domain” were not carried forward and retained at that level. The 2017 standards added two competencies (Universal Competencies) to the domain level that speak to the attributes of the practitioner. Namely “practicing professionally” and “communication, collaboration and leadership”.

Both standards included many similar domains and sub-domains with the 2017 standards being considerably less detailed. This was best illustrated in radiography / radiology. The 2009 standards (Element 6.4, 6.6 and 7.1) contain 10 times the number of performance criteria (3 versus at least 35) in the 2017 standards (Competency 3.3 and 3.5). The statements in the 2017 standards are “demonstrates knowledge of diagnostic imaging techniques and procedures, including indications and limitations of available imaging modalities (3.5.1)”, “refers for or conducts imaging where clinically relevant (3.3.4)” and “identifies existing investigation results and reports (3.3.1)”. Whereas the 2009 standards contain 11 performance criteria for radiographic technology (6.4) and 14 for radiographic technology.

#### Added to the 2017 graduate competencies

The construction of the table to matching both standards revealed 18 additions to the 2017 graduate competencies that were not present in the 2009 version (See Table 2). Summary lists are also provided to enable easier analysis (See Table 3). Additions were noted that resonated with contemporary issues of patient-centred care as exemplified by respect for practitioner and patient boundaries, patient sexual orientation, confidentiality, and agreement from the patient on care and the transition to self-management with improved lifestyle options.

The 2017 graduate competencies also had added the performance criteria an expectation to be self-aware of their personal biases and beliefs, factors that impact on their own health, meet language proficiency requirements, avoid discipline specific language, and seek professional support and peer review when necessary. In addition, they are expected to be effective users of information technology to enhance their practices, actively applying principles of risk management and quality improvement. Also, the 2017 graduate competencies include the expectations to provide timely and sensitive feedback to colleagues, recognise the potential for disagreement and conflict in relation to care and management and respond to resolve these issues.

With respect to clinical expectations the 2017 graduate competencies have added identifying and managing “Red flags” and chronic conditions as well as monitoring patients progress towards achieving pre-planned health outcomes using validated and reliable measures.

#### Not carried forward from the 2009 to 2017 graduate competencies

The 2017 graduate competencies no longer contain the requirement to have an awareness of chiropractic’s professional special characteristics, aspirations and major historical milestones or having knowledge of the major professional organisations. No longer required is an understanding of the relevant health care economics.

There is a change in the use of terms such as “differential diagnosis” which is replaced with “clinical impression”, and “chiropractic techniques” has been broadened to “adjustive, manipulative, manual and other therapies” and “contra/non/indications” is replaced with “therapeutically effective”.

With respect to the physical environment, there is no longer the need for being competent in business, staff and financial management. This includes the specific detail of practice furniture, colour coding, music, temperature control or training of the staff to maintain “an environment of unconditional positive regard”.

For the clinical encounter, the graduate is no longer expected to discuss with the patient the cost of care, an appropriate discharge point, nor re-evaluate and monitor at each visit. Removed also is the need to demonstrate the ability to measure impairment, disability and handicaps, use screening instruments for the most common mental health disorders, and ensure adequate, on-going care for patients in times of absence.

Removed also is the details for radiographic technology. Directives for the manner of conducting a physical examination such as pursuing abnormal findings in a deliberate, logical and appropriate manner and assessing the reliability of the data obtained and its correlation with that of the patients’ complaints no longer exist. Finally graduates are no longer expected to demonstrate a willingness and capacity for writing third party and medicolegal reports certificates and correspondence.

### Word analysis / frequencies (Tables 4)

The 2017 CCE-Australasia standards are over 3 times smaller than the 2009 standards for graduating chiropractors. Consequently, at a minimum, a key word should be at least 3 times more or less frequent to warrant inclusion in this section of the analysis.

**Table 4:** The frequency of key words (or their derivatives) in the 2009 and 2017 CCE-A competency standards for graduating chiropractors

	2009 CCEA Graduate Competencies			2017 CCEA Graduate Competencies		
Word	Total number	Heading	Perf Criteria	Total Number	Heading	Perf Criteria
Accountability	2	2		1		1
Adequate	16	4	12	0		
Advice	0			1		1
Applies	8	4	4	9	2	7
Appropriate	45	4		5		5
Assessment	23	5	18	17	2	15
Awareness	23	7	16	2		2
Bases	2	2		0		
Care/ing/ful	46	13	21	35	14	21
Clearly	11	11		0		
Clinical	33		33	13	6	7
Collaboration	3	3		7	2	5
Communicate/ion	17	4	13	10	4	6
Community	4	2	2	5		5
Competent	9	7	9	5	2	3
Competence/y	7	7		52	28	24
complies	1			1		
Confidentiality	0		0	3		3
Consent	5	2	3	2	1	1
Consider	19	1	18	5	2	3
Consultation (ed)	7		7	6	5	1
Contra-indication	6		6	0		
Counsels	7	4	3	0		
Critically	1		1	3	1	2
Data	26	2	24	0		
Define	1		1	1		1
Demonstrate	11	2	9	14	2	12
Development	11	6	5	5		5
Diagnosis/tic	28	5	26	5	1	4
Disease	19	6	13	2		2
Effective	21	8	13	15	5	10
Ethical	8	2	6	9	3	6
Evaluate	21	2	19	2	1	1
Evidence-based	1		1	9	3	6
Examination	16	2	14	5	1	4
Explains	20	2	18	4	1	3
Factors	9		9	4		4
Family	13		13	0		
Finances	2		2	0		
Goal (s)	2		2	1		1
Healthcare	14	6	8	0		
History	14	4	10	3	1	2
Imaging	1		1	3		3
Improvement	1		1	7	2	5
Indicator/ed	41	39	2	5		5
Information	13	4	7	7	2	5
Integrity	1		1	1		1
Interprets	8	5	3	2		2
Inter-professional				1		1
Knowledge	10	3	7	15		15
Leadership	0			3	3	
Lifestyle	0			1		1
Limit / ations	13	3	10	2		2
Management	23	14	9	24	3	21
Manner	21		21	2		2
Measure	1		1	3		3
Obtains	4	2	2	8	4	4
Options	3		3	12	3	9
Outcome	0			8	2	6
Patient	130	19	111	40	6	36
Patient-centre	0			3		3
Participate/ion	3		3	21		2
Perform	51	40	11	25	23	2
Personal	12	2	10	4	1	3
Physical	26	4	22	1		1
Plan	14	5	9	9	5	4
Practice	19	6	13	25	9	14
Prevention	7	5	2	3	1	2
Problems	13		13	1		1
Procedure	31	1	30	6	1	5
Professional	30	5	25	43	5	38
Promote (ion)	7	2	5	3	1	2
Provider	13	8	5	7	2	5
Public	10	2	8	5	1	4
Quality	3		3	15	3	12
Radiographic	16	2	14	0		
Recognize	14		14	9	1	8
Records	17	4	13	5	2	3
Referral	13	4	11	5		5
Relevant	20	2	18	10	2	8
Research	1		1	5		5
Responsible	2		2	2		2
Requirements	5		5	5	1	4
Risks	5		5	2		2
Safe / ty	9	3	6	19	4	15
Scope of Practice	0			0		
Selection	4		4	0		
Self-management	1		1	1		1
Skills	5	2	3	9		9
Staff	11	6	5	3	1	2
Stakeholders	0			5		5
Standard	5	4	1	10	7	3
Status	10		10	8		8
Strategies	1		1	1		1
Study	4		4	7	2	5
Subluxation	0			0		
Support	5		5	0		
Treatment	5	2	3	3		3
Understands	14	4	10	2		2
Vitalism	0			0		

**Fig. 1:**
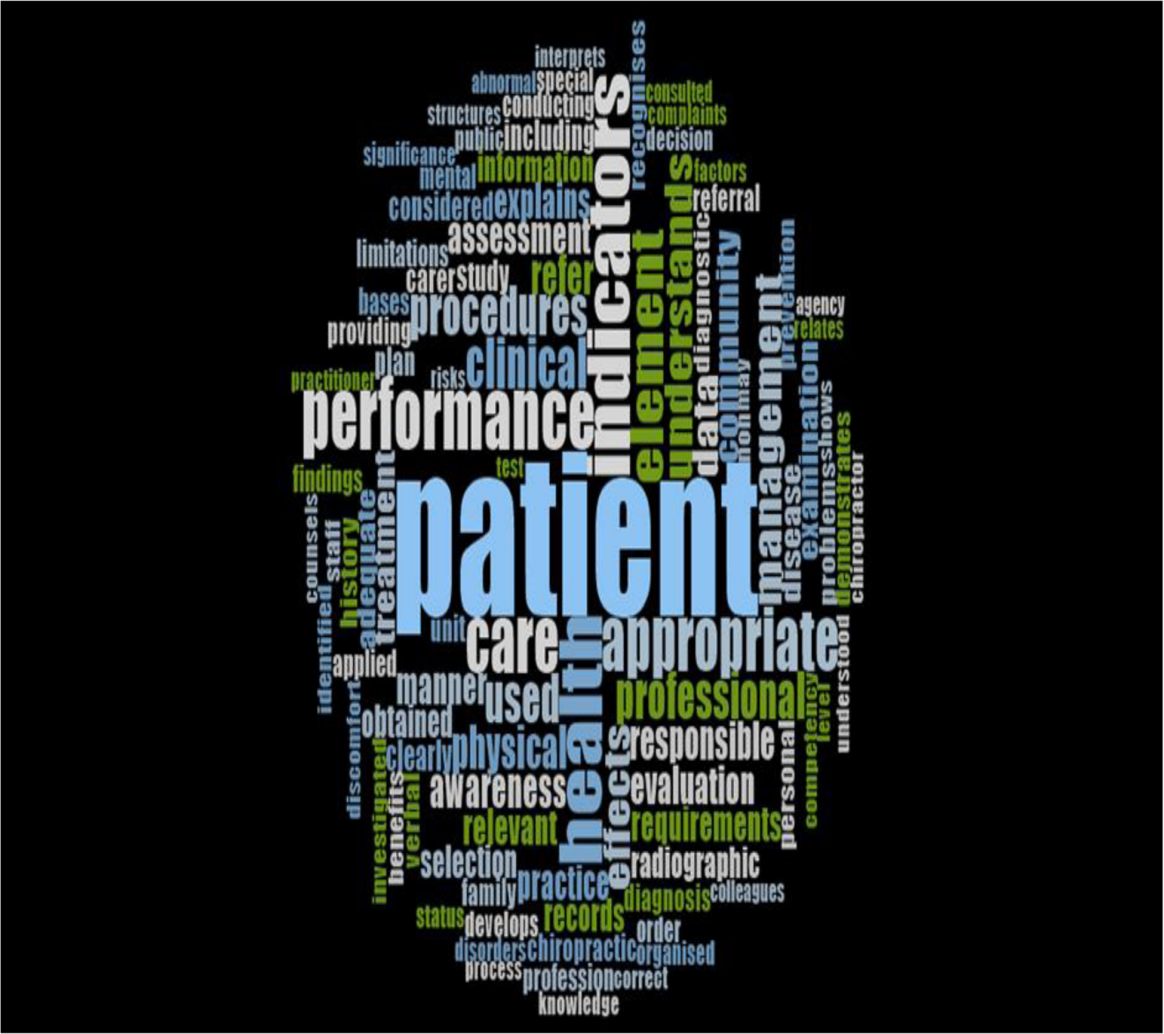
Word Cloud for CCE-A 2009 competency standards for graduating chiropractors created from Nvivo

**Fig. 2:**
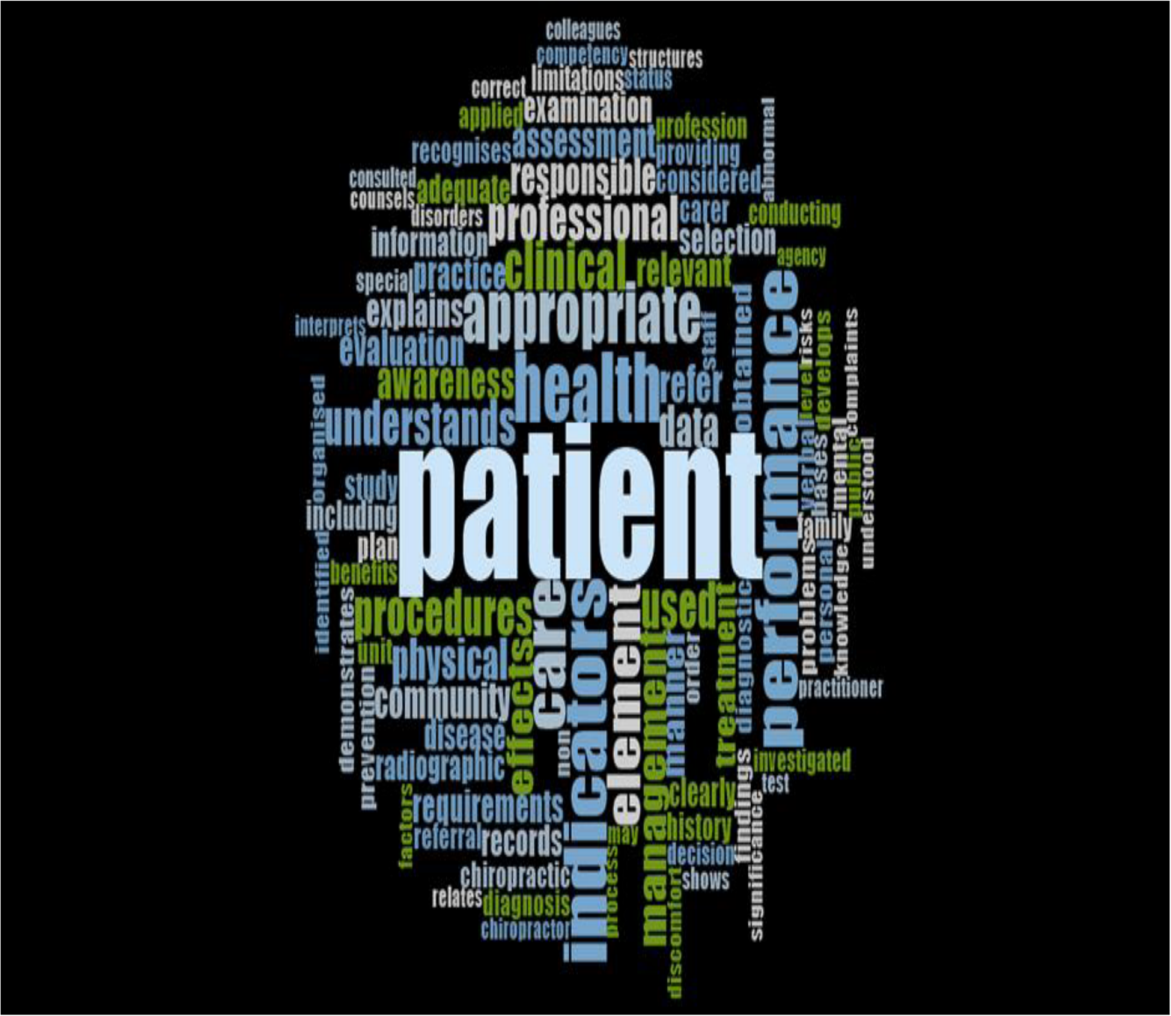
Word Cloud for CCE-A 2017 competency standards for graduating chiropractors created from Nvivo

Somewhat unsurprisingly the most frequent word was “patient”, occurring 130 times in the 2009 standards and 40 in the 2017 standards (see Tables 4). This is evident in the Nvivo created word clouds (Figs. 1 and 2). The word chiropractic/or was also very frequent. In the 2017 graduate competencies this is mentioned 99 times. Of these 18 were footnotes, 10 as part of organisational title e.g., CCEA, 5 as headings, and as text 66 times. The 2009 standards although 3 times larger use these words 4 times less (21 times). Once in an organisational title, 9 as headings, and 11 in text.

Words that increased in frequency in the 2017 graduate competencies, despite being smaller, that indicated a move toward a contemporary mainstream health care approach were collaboration (3 in 2009 versus 7 in 2017), evidence-based (1 vs. 9), professional (30 vs. 43), patient-centred (0 vs. 3), and research (1 vs. 5).

Increased numbers of words that indicate a changed emphasis on the quality of patient care were competence/y (7 in 2009 vs. 52 in 2017), ‘confidentiality’ (0 vs. 3), ‘demonstrate’ (11 vs.14), ‘safety’ (9 vs. 19), ‘skills’ (5 vs. 9) and ‘quality’ (3 vs. 15).

Words that indicated a more integrated role for chiropractors in the health care system in the 2017 standards were ‘community’ (4 in 2009 vs.5 in 2017), ‘collaboration’ (3 vs. 7), ‘inter-professional’ (0 vs. 1), and ‘stakeholders’ (0 vs. 5).

Words that suggest a more outcomes-based approach to accreditation of CPs were ‘outcomes’ (0 vs. 8), ‘performance’ (51 vs.25), ‘demonstrate’ (11 vs. 14), and ‘effective’ (21 vs. 15).

There appears to be increased expectations for the quality of some practitioner behavioural characteristics for the graduating practitioner as seen by increases in the words ‘communication/ing’ (17 vs. 10), ‘competence/tent’ (7 vs. 52), ‘ethical’ (8 vs. 9), ‘leadership’ (0 vs. 3), and ‘practice’ (19 vs.25).

Other descriptive words were considerably reduced beyond the 3 to 1 ratio from 2009 to 2017 were ‘data’ (26 in 2009 vs. 0 in 2017), ‘adequate’ (16 vs. 0), ‘appropriate’ (45 vs.5), ‘awareness’ (32 vs.2), ‘diagnosis/tic’ (28 vs. 5), ‘disease’ (19 vs. 2), ‘clearly’ (11 vs. 0 in 2017), ‘contraindication’ (6 vs. 0), ‘counsels’ (7 vs. 0), ‘evaluate’ (21 vs. 2), ‘radiographic’ (16 vs. 0), ‘selection’ (4 vs. 0), ‘staff’ (11 vs. 3), ‘support’ (5 vs. 0), and ‘understands’ (14 vs. 2).

## Discussion

### Objective 1: summary of findings

This is the first study to explore changes in CCE accreditation standards for graduating chiropractor competencies over time for indicators of progressive change.

The 2017 and 2009 standards are similar in that they share the same broad descriptive framework for describing the practitioner-patient interface by using the chronology of the consultation process. The new standards have provided less descriptive information on nearly all the entry-level graduate competencies areas and moved toward using broader descriptive terms of expected behaviours. The construction of the standards was more transparent and described the consultation process and sought feedback from a wide range of stakeholders.

We found that CCE-A competency standards of 2017 have moved in a positive direction and embraced standards of other widely accepted allied-health professions by adding expectations for managing chronic conditions, lifestyle issues, and embraced patient-centred care and self-management. However, some differences and omissions were not positive. Omissions included some of the prescriptive detail around clinical practice being removed. For example, chiropractors are no longer expected to discuss with the patient the cost of care, screen for mental health conditions, an appropriate discharge point, nor re-evaluate and monitor at each visit. Continued silence was noted on the contemporary issue of the need for a clear definition of “chiropractic/or”, and on the presence of “vitalism” and “subluxation” in CPs.

### Objective 2: discussion of findings

#### Construction of accreditation standards

Concerns have been raised about the lack of transparency for initiatives and changes being adopted by accreditation agencies [28–30]. The current CCE-A standards addressed many of these issues by undertaking a review process, inclusion of many ‘stakeholders’, an “environmental scan” and a consultation process that reviewed responses and made amendments and additions. However, the criteria for those amendments / changes are not explained.

Recent studies exploring accreditation standards and processes of chiropractic programs have raised questions about the absence of an evidence-based approach [9, 10, 13]. Some have suggested that the improvement of accreditation standards has been assumed and not measured [3]. Medical studies exploring changes to accreditation standards have shown that they should involve a review of the evidence base for each standard as well as a trial “in the field” for the opportunity to further refine them [28]. It is unfortunate that no information was provided in the CCE-A standards on the extent of the review of the evidence base for each standard or if there was a “field trial”. A follow-up analysis would have indicated if the impact of the new standards was as intended [21].

Part of a “field trial” would be to determine if the language contained within the standards was interpreted by everybody in the same way [31]. If a term is understood differently then it becomes difficult to create a shared agenda, common goals, or to monitor changes [32]. Definitions are viewed by some as the starting point for reform [33, 34]. The obvious omission in these competencies is the absence of a definition of chiropractic or chiropractor in either set of graduate competencies [32, 35]. It has been shown that the desire to accommodate a diverse range of intra-professional understandings of chiropractic has resulted in the absence of a detailed professional definition [36, 37]. Patients want a practitioner who deals with musculoskeletal issues [38] and economies require a practitioner who can better manage spinal pain [2]. A definition of chiropractic and its attendant scope of practice based on these outcomes would better inform the construction of competencies for entry-level graduates to this end and should therefore be part of future accreditation standards [35].

#### Graduate competencies

There are many encouraging signs when looking at the additions to the 2017 graduate competencies that reflect contemporary mainstream health care standards. The frequency of the term “evidence-based” has been shown to be an indicator of the quality of accreditation standards and their regulation [24, 39] and this has dramatically increased in 2017 competencies. The competencies are clearly patient-centric and embrace contemporary issues of sexual orientation and patient boundaries. Also, they appear to be mindful of the economic impact of the disability resulting from LBP with specific requirements for transition to self-management, managing chronic conditions and improving lifestyle options all of which should be monitored with valid and reliable measures [5, 40, 41].

There have been concerns over the lack of quality measures available to regulators for assessments of some of the stipulated standards [42]. There appear to be competencies in the 2017 standards that will be very difficult to establish criteria or measures to ensure compliance. An example within these competencies would be measuring if a CPs graduates have met the requirement of being “self-aware of their personal biases and beliefs”. Another example is the requirement for graduates to be “seeking peer review when necessary”. For this competency there is no information surrounding what is appropriate peer review and under what circumstances it may be required. A search of the available literature in Pubmed, Scopus and Index to Chiropractic Literature failed to find any studies for the application of peer review in chiropractic practice that might inform this requirement.

Some competencies omitted are less likely to impact on patient safety and quality of care such as those for practice management of ensuring office colour coding and music. Others appear to be changes in language applied to the same competency such as the recognition that chiropractic techniques now involve adjustive, manual and other therapies and therapeutically effective encompasses contra/non/indications.

Other competencies that were omitted are less easily passed by. Of concern was the competent ability to discuss cost of care, the ability to re-evaluate at each consultation and appropriate discharge. Also, the ability to use screening instruments for common mental health disorders which are a growing area of public health concern [43], and an important co-morbidity in disabling persistent pain suffers [44] is more troubling.

The use of radiography in chiropractic care is an ongoing debate within the chiropractic profession [45, 46]. At first glance the 2017 competencies of “refers or conducts imaging when clinically indicated and knowledge of diagnostic imaging indications and limitations” appears reasoned. Some state that the clinical indication for the use of imaging is for detecting contra-indications to spinal manipulation as well as identifying spinal lesions (subluxations) [47, 48] and this sits uncomfortably with current guidelines for its use in musculoskeletal practice [49, 50]. The dramatic reduction in the amount of prescriptive graduate competencies from 2009 to 2017 does little to resolve this issue. The current language appears to allow both clinical rationales to co-exit under the same competency and may require further detail for clarification for chiropractic educators and programs going forward.

#### What is not in the CCE-Australasia 2017 and 2009 competency standards

There are known and documented relevant issues within the chiropractic profession that have not appeared within either of the competency standards. One such area is the inclusion into chiropractic curriculums of non-evidence-based constructs such as *subluxation* as an ‘objective’ lesion and *vitalism* as a model of treatment other than as a historical concept [24, 51]. Chiropractic programs that actively teach *vitalism* have been shown to be related to practitioners who are more likely to consider the chiropractic *subluxation* as an encumbrance to the expression of health, anti-vaccination attitudes, and low levels of inter-professional referrals [18]. There is contemporary evidence that shows this is still the case in some chiropractic institutions even though they remain accredited [52, 53]. A definition of chiropractic practice may go some way to addressing this absence. Silence on this matter does little to assist the integration of chiropractic into the wider health care community and in our opinion requires addressing.

#### Methodological considerations

The methodology used in this study was very similar to that comparing sequential versions of the accreditation standards of the CCE-International [22] and therefore shares many methodological considerations. Namely a confidence that this has resulted in a comprehensive comparison and matching of the areas, subareas and terms used in the 2009 and 2017 CCE-A Competency Standards. Also that the search methodology and word count for key terms could have resulted in other findings or conclusions [25]. Further studies of this nature will likely confirm if this is the case. In support of this confidence is the high level of agreement between coders for theme identification, text interpretation and allocation that did not require a third author for disputation purposes.

We are aware that chiropractic program evaluation involves several facets that extend beyond graduate competencies alone. This includes the self-evaluation report, the site inspection and the quality review process. However, as these standards are intended to guide chiropractic programs they become important documents to scrutinise and critique.

### Objective 3: recommendations

This review has sought to identify similarities and differences between revisions of the CCE-A graduate entry level competencies. This has led to the identification of a number of issues and, based on these, we make a number of recommendations that are summarised in Table 5. If these recommendations were adopted, then CPs would better understand the expectations of the CCE-A and educators could amend their curriculum appropriately, as well as provide measures to demonstrate compliance and quality improvements. The intended end result being practitioners entering the workforce who can better deliver ethical, safe and quality care.

**Table 5:** Summary of Recommendations

	Recommendation	Justification
**1**	Create a clear definition of chiropractor.	This lays the foundation for competency development, scope of practice, and limits confusion on professional identity.
**2**	A review of the impact of the changed competency standards.	To ensure they are achieving the desired outcomes.
**3**	Greater descriptive material for competencies, eg., peer review, self-aware of personal biases.	To allow for reliable and valid assessment of achievement by CPs.
**4**	Review of competencies omitted from 2017 for inclusion in future revisions. Eg., re-evaluate & monitor at each consultation, appropriate discharge, use screening instruments for common mental health disorders.	This information is not provided in the current standards and could enhance the quality of patient care and safety.
**5**	Targeted descriptive information surrounding areas of known difficulty within chiropractic. Eg., radiology and *vitalism/subluxation.*	Bring the curriculum of CPs in line with other contemporary mainstream allied healthcare professions.

## Conclusions

The CCE-A Competency Standards, while less detailed, appear to be moving in a direction that is in accord with mainstream allied healthcare. They are more transparent about their development, engaged with a wide range of stakeholders and increased the use of contemporary healthcare language. Some differences and omissions were not positive and reflect on-going intra-professional issues such as the lack of definition of chiropractic and an absence of discussion of “vitalism” and “subluxation”. Silence on these matters does little to address the confusion among the public, other health professionals as to an appropriate scope of practice. The removal of the need to be competent in re-evaluation at each consultation, appropriate discharge and screening for mental health conditions have the potential to impact on patient safety and quality of care. These matters appear to require redressing in future iterations of CCE-A Competency Standards.

## Data Availability

Not applicable.

## Abbreviations

CCE: Council on Chiropractic Education
CCE-A: Council on Chiropractic - Australasia
CPs: Chiropractic Programs
